# Dental caries status of Bulang preschool children in Southwest China

**DOI:** 10.1186/1472-6831-14-16

**Published:** 2014-03-04

**Authors:** Shinan Zhang, Juan Liu, Edward CM Lo, Chun-Hung Chu

**Affiliations:** 1 Faculty of Dentistry, The University of Hong Kong, Hong Kong, China; 2 School of Stomatology, Kunming Medical University, Kunming, Yunnan, China

**Keywords:** Caries, Children, Ethnic, Minority, China

## Abstract

**Background:**

Bulang is one of the 55 ethnic minorities in China with a population of around 120,000. They live mainly in Yunnan, which is a less-developed province in southwestern China. Many Bulang people live in remote villages and have little access to dental care. They like hot and sour food and chew betel nut. This study examines the caries status of 5-year-old Bulang children and factors that influence their caries status.

**Methods:**

A sample of 5-year-old Bulang children in Yunnan was selected using a multi-stage cluster sampling method. One trained dentist examined the children using dental mirrors with intra-oral LED light and CPI probes. Caries experience was measured according to the dmft index. Oral hygiene status was recorded according to the visible plaque index (VPI). A parental questionnaire was used to study the children’s oral health-related behaviours.

**Results:**

A total of 775 children were invited and 723 joined the survey. The caries prevalence was 85%, and 38% of them had caries involved in pulp. The mean dmft and dt score were 5.8 ± 4.9 and 5.6 ± 4.8, respectively. Visible plaque was found on 636 children (88%). Multi-factor ANCOVA analysis found that higher dmft scores were found among the children who snacked on sweets daily, had visited a dentist within the last year and had higher VPI scores.

**Conclusions:**

The caries prevalence and experience among 5-year-old Bulang children in Yunnan was high, and most of the caries were left untreated. The caries experience was associated with snacking habits, dental visit habits and oral hygiene.

## Background

Although dental caries is well understood and preventable, it is still a serious global problem among children [1]. Dental caries causes pain, impairs eating and sleeping and causes local and systemic infections [2]. American children missed an estimated 51 million school hours because of dental caries in 2000 [3]. The Fédération Dentaire Internationale (FDI) World Dental Federation’s *Oral Health Atlas* estimates that untreated dental caries affects more than half of the children in most Southeast Asian countries [3]. In the Philippines, the main reason for absence from school was dental caries [4]. There is overwhelming evidence that dental caries is unequally distributed and is exceptionally high among ethnic minority groups [5].

Ethnicity has been considered a marker of oral health status [5]. The members of an ethnic group share a pattern of actions or beliefs that can influence their oral health practices, such as diet, care-seeking behaviours, using of home remedies, values placed on having healthy primary teeth or expectations about preventive or therapeutic interventions [6]. The impact of the disparities associated with ethnicity is becoming increasingly significant and elimination of the health and health care disparities based on ethnicity is a priority in many countries.

There are 55 ethnic minority groups in China and their population is approximately 114 million [5]. They are scattered across the country, but the majority live inland or in border districts in the less-developed western region. These ethnic minorities are culturally and linguistically diverse. Some ethnic minority groups such as Zhuang and Hui have more than 10,000,000 people, whereas some ethnic groups have fewer than 100,000. Bulang is one of the ethnic minority groups with a relatively small population (around 120,000) [5]. Most of the Bulang (97%) live in the mountainous areas along the Mekong River, on the southwestern frontiers of Yunnan Province 1,500 to 2,300 metres above sea level. The social development of the Bulang people is below the national average. It is one of the seven poor ethnic minority groups that receive special economic support from government [7]. Outside of China, Bulang people live in the neighbouring countries of Myanmar, Laos and Thailand.

Bulang people speak a language that belongs to the South Asian language family. The language has no written form. The Bulang people in China often live among other ethnic groups and most of the young people can understand Mandarin (official language in China). Rice, maize and beans are their staple foods and they like sour and hot food. Drinking home-brewed wine and smoking tobacco are their pastimes. Bulang women chew betel nut and regard teeth stained black with betel-nut juice as beautiful [8]. Bulang people live in areas that produce cane sugar. The availability of locally produced sugar (sucrose) in various forms, such as sweets and soft drinks, imposes an increased caries risk to children. In addition, there is no water fluoridation in China [9], and access to dental care is very limited. This could impose a high caries risk to Bulang children. Moreover, there is little access to dental care since they live in remote, mountainous areas.

The authors of this paper have been conducting epidemiological surveys to study the caries status of 5-year old preschool children aiming to help map out the caries prevalence and the underlying factors for this in a range of ethnic populations in South East Asia, including Southern Chinese children [10], Cambodian children [11], Pilipino children [12], Myanmar children [13], Wa children [14] and Dai children [15]. This study followed the same survey method of these previous studies to assess the caries status of 5-year-old Bulang children. The literature review found only one study in 1994 reporting caries among 6-year-old Bulang children [5,16]. The study used a convenience sample of 122 children and did not investigate determinants of the children’s caries status. This study aims to examine the dental caries status of and investigate the factors that affect the dental caries status of five-year-old Bulang children in China. It is considered to be a part of a series of surveys aiming to map out the oral disease prevalence and the underlying factors for caries in a range of ethnic populations in South East Asia.

## Methods

### Selection of children and sample size

This study was approved by the Institutional Review Board of the University of Hong Kong (IRB UW-11-377). The main study was performed from November 2011 to May 2012. Since the diagnostic criteria of caries in the 1994 survey were not at cavitation level [16], a pilot study using a convenience sample was performed on 127 five-year-old children in Lincang, Yunnan where Bulang people reside. The caries prevalence was 81%. With a confidence interval set at 3%, the sample size required in this survey would be 684. The response rate was estimated to be 90%, and as such, this study aimed to recruit at least 760 children.

Bulang people are scattered but mainly live in the southwestern rural areas of the Yunnan province. A multi-stage cluster sampling method was used according to the geographic distribution and resident areas of Bulang people [8]. In the first stage, two districts (Xishuangbanna and Lincang) with the largest Bulang population were selected. The 2 districts account for the majority (77%) of the total Bulang population in Yunnan. At least 380 5-year-old Bulang children (50% of the sample size) from each of the 2 selected districts were invited. In the second stage, the counties that account for the majority (more than 50%) of Bulang people were selected from the district. Therefore, 2 of the 3 counties in Xishuangbanna (Jinghong and Menghai) and 3 of the 8 counties in Lincang (Yun, Gengma and Shuangjiang) were selected. All kindergartens and preschools (which provide early childhood education) in the town and villages of the selected counties were invited to join this survey. Since preschool education is not compulsory in China, 5-year-old children who did not attend kindergarten or preschool were invited with the help support of the Bureau of Public Health, Bureau of Education of the local government and leaders and elders of the villages. They helped in the recruitment of the 5-year-old children and their parents in this study.

The invitation letters and consent forms were given to the parents of the children by the teachers of the participating kindergartens and preschools. Leaders and elders of the villages and towns also helped to contact the parents of the children and encouraged them to join the study. The protocol of the study was explained to these parents, and their written informed consent was obtained before the study. Children in the village and not attending kindergarten or preschool were invited for examination in the community centre of the village. Children who had major systemic diseases or syndromes or who were on long-term medication were excluded from the study. The study that consisted of a questionnaire survey and a clinical examination was conducted in the kindergartens, preschools and community centres. A trained dentist performed the clinical examination. There were 3 other helpers: one did registration, one performed charting and one local person who spoke the dialect assisted the parent in completing the questionnaire survey.

### Questionnaire survey

A self-completed questionnaire (attached as Additional file 1) that was used in a previous survey for a minority of 5-year-old Dai children in China was distributed to the parents of the children [15]. The parents who brought the children were asked to complete the questionnaire on the spot the same day before the clinical examination of their children. They were asked to complete the questionnaire that included two parts:

1. Demographic information – child’s age, sex, education level of parents, caretaker (parent as main caretaker), home place (town or village);

2. Oral health-related behaviours – child’s snacking habit (snacking on sweets daily), bottle-feeding habit (bottle feeding before sleep), tooth-brushing practice (daily tooth brushing) and dental visits (visited a dentist in the last year).

An assistant who spoke local dialect was available to help them fill in the questionnaire. The completed questionnaires were immediately collected by a research assistant on site.

### Clinical examination

All the children received a clinical examination conducted by a trained dentist in kindergartens, preschools, or community centres. Dental caries status was accessed using criteria recommended by the World Health Organization [17]. Caries was diagnosed at cavitation level mainly by visual inspection using a disposable dental mirror attached to an intra-oral LED light [17]. A 0.5 mm ball-ended CPI probe was used to remove debris and to confirm the presence of the cavity.

The dmft index was used to record the caries experience of the primary dentition. The clinical consequence of caries disease was measured by the “pa” index, which was modified from the pufa index [18]. The “p” stands for an untreated carious tooth with visible pulp involvement, and the “a” denotes an untreated carious tooth with visible apical infection that can be in the form of abscess or fistula. Oral hygiene status was measured using the Visible Plaque Index (VPI) [19]. The presence of clearly visible plaque on the buccal surfaces of 6 index primary teeth (55, 53, 51, 71, 73 and 75) was recorded; and the percentage of the 6 examined sites with visible plaque was calculated to represent the oral hygiene status of the child. Duplication examinations were performed on 10% of the children. The Kappa statistic was used to assess the intra-examiner reproducibility.

### Data entry and analysis

The data collected were entered into a personal computer and analysed using software IBM SPSS Statistics 20. Cohen’s Kappa was used to assess the intra-examiner agreement on the assessment of caries status (dmft), clinical consequence of caries disease (pa) and oral hygiene status (VPI). Descriptive statistics were produced to report the caries status, the percentage with clinical consequence of caries disease and oral hygiene status. The independent variables included sex, education level of parents, caretaker, home place, child’s snacking habit, bottle-feeding habit, tooth-brushing practice and dental visits. Comparisons were made using an independent *t*-test (2 categories) and one-way ANOVA (more than 2 categories) to assess the statistical significance of the differences in the dental caries experience (mean dmft scores). Multiple comparisons using the Bonferroni test was performed to compare the groups (N > 2) when the independent variable was found to be a significant factor affecting the caries experience (mean dmft scores). A Chi square test was used to compare the caries prevalence between anterior teeth and posterior teeth and between maxillary teeth and mandibular teeth. Analysis of covariance (ANCOVA) was also performed to identify the socio-demographic and behavioural determinants of dental caries experience (dmft scores) on the surveyed children. All the independent variables were entered into the model. Backward stepwise procedure was performed until only variables demonstrating a statistically significant association remained in the final model. The statistical significance level for all tests was set at 5%.

## Results

The survey was carried out in kindergartens, preschools and community centres of the selected counties of the 2 districts. This study invited 775 Bulang 5-year-old children, of whom 723 (365 boys and 358 girls) from 19 kindergartens, 16 preschools and 31 community centres completed the oral examination. Around half the children (352) were from community centres. There were 203 (28%) children who attended kindergarten and 23% (N = 168) attended preschool. The response rate was 93%. Among those who did not attend the clinical examination, 44 children were absent on the day of examination, and 8 did not cooperate with the examination. The Kappa’s values for the assessment of caries (dmft), clinical consequence of caries disease (pa) and oral hygiene (VPI) were 0.98, 0.97 and 0.95, respectively.

Table 1 shows the caries experience, caries prevalence and the clinical consequence of caries disease and its prevalence amongst the Bulang 5-year-olds. Eighty-five per cent of the children had caries. The mean (SD) dmft was 5.8 (4.9). Most of the caries found were left untreated. The mean (SD) dt score was 5.6 (4.8). There were 275 children (38%) who had at least one carious tooth with pulp involvement (pa > 0), and their mean (SD) pa was 1.2 (2.3). Visible apical infection in the form of abscess or fistula (a > 0) was found in 96 children (13%).

**Table 1:** Caries experience (dmft) and clinical consequence of caries disease (pa) of Bulang children (N = 723)

**Dental caries**	**Prevalence**	**Mean (SD)**
Caries experience (dmft)	85%	5.8 (4.9)
Decay teeth (dt)	84%	5.6 (4.8)
Missing teeth due to caries (mt)	6%	0.1 (0.6)
Filled teeth (ft)	1%	<0.1 (0.4)
Prevalence of severe caries – pulp involved (pa)	38%	1.2 (2.3)
Visible apical infection – abscess or fistula (a)	13%	0.2 (0.5)

**Figure 1:**
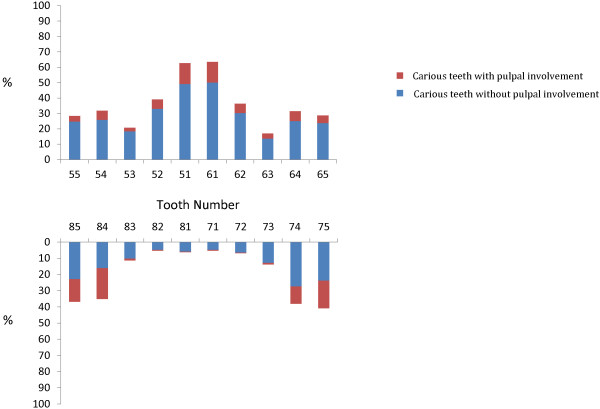
Untreated caries prevalence (%) and tooth number.

The prevalence of caries was highest among the maxillary primary central incisors (66%) and lowest among the mandibular primary incisors (5%) (Figure 1). The association of caries prevalence and tooth type is shown in Table 2. Mandibular primary molars had more caries than their maxillary counterparts (60% vs. 54%, p < 0.001), and many mandibular primary molars had advanced caries with pulpal involvement (28%). Seventy-one per cent of the children had caries in their maxillary anterior teeth, and this was found to be associated with the prevalence of caries in their posterior teeth (p < 0.001).

**Table 2:** Caries prevalence according to the tooth type

	**Anterior (Incisors and Canines)**	**Posterior (Molars)**	**p-value**
All	522(72%)	497(69%)	<0.001
Maxillary arch	514(71%)	388(54%)	<0.001
Mandibular arch	145(20%)	432(60%)	<0.001
p-value	<0.001	<0.001	

Visible plaque on the index teeth was found in 636 children (88%) and 42 children (6%) had visible plaque detected on all 6 index teeth. The mean (SD) VPI score was 42% (28%). A multiple regression analysis showed that higher VPI scores were found among children who lived in towns and were not cared for by their parents (Table 3).

**Table 3:** Relationship between oral hygiene status (VPI) and selected independent variables (final model of multi-factor ANOVA)

**Independent variables**	**Group**	**Beta**	**SE**	**Eta-squared**	**p-value**
Site	Towns Villages^a^	0.084	0.021	0.001	<0.001
Visited a dentist within last year	Non-parents	0.072	0.026	0.027	0.005
	Parents^a^				
Intercept		0.360	0.015		<0.001

^a^Reference Category, Adjusted R^2^ = 0.033.

Table 4 shows the relationship of dental caries experience and variables studied using an independent sample *t*-test. Locations (village or town), father’s and mother’s education, care by parents, tooth-brushing habits and history of dental visits were found to be significant factors affecting the caries experience of the children (p < 0.05). All the variables studies and VPI score were entered to build a multivariable model using covariance analysis (ANCOVA). The results showed that higher dmft scores were found among the children who snacked on sweets daily, had visited a dentist within the last year and had a higher visible plaque score (Table 5). Among the children with caries, those who snacked on sweets daily, visited a dentist within last one year and had a high visible plaque score had more teeth with clinical consequence of caries disease.

**Table 4:** Caries experience (dmft) and variables studied

**Variables (%, N)**	**dmft (SD)**	**p-value (Bonferroni Comparison)**
Sex		
Boys (51%, 365)	5.8 (4.9)	0.776
Girls (49%, 358)	5.7 (4.9)	
Location		
Towns (47%, 337)	6.4 (5.0)	0.029
Villages (53%, 386)	5.2 (4.7)	
Father’s education		
Below secondary^a^ (64%, 434)	4.6 (4.1)	
Secondary^b^ (24%, 166)	5.5 (4.2)	0.016
Tertiary or above^c^ (12%, 82)	5.7 (4.7)	(a < b, c)
Mother’s education		
Below secondary^a^ (71%, 481)	4.7 (4.1)	
Secondary^b^ (18%, 125)	5.8 (4.5)	0.033
Tertiary or above^c^ (11%, 76)	5.0 (4.3)	(a < b, c)
Parents as main care-taker		
Yes (77%, 528)	4.6 (4.2)	0.002
No (23%,154)	5.8 (4.3)	
Bottle feeding before sleep		
Yes (13%, 85)	5.4 (4.4)	0.219
No (87%, 592)	4.8 (4.2)	
Snacking on sweets daily		
Yes (69%, 468)	5.1 (4.3)	0.072
No (31%, 206)	4.5 (4.2)	
Daily tooth brushing		
Yes (37%, 250)	5.4 (4.6)	0.019
No (63%, 430)	4.6 (4.0)	
Visited a dentist within last year		
Yes (10%, 68)	8.1 (4.6)	<0.001
No (90%, 612)	4.6 (4.1)	

**Table 5:** Relationship between dental caries experience and selected independent variables (final model of multi-factor ANCOVA)

**Independent variables**	**Group**	**Beta**	**SE**	**Eta-squared**	**p-value**
Snacking on sweets daily	Yes	0.509	0.241	0.002	0.035
	No^a^				
Visited a dentist within last year	Yes	1.296	0.377	0.005	0.001
	No^a^				
VPI		10.628	0.402	0.506	<0.001
Intercept		0.022	0.257		0.931

^a^Reference Category, Adjusted R^2^ = 0.541.

## Discussion

A recent review concluded that caries experience and prevalence among preschool children was high in China [5]. Oral health is an integral part of general health. However, the oral health status of the ethnic minority children was not routinely reported in China in the past national oral health surveys. Many ethnic minorities live in mountainous or isolated areas. Conducting an oral health survey for them can be methodologically and logistically demanding. Thus, reports of oral health of ethnic minorities are few even though they are essential in the planning and implementation of a dental health promotion programme [20].

This study required a relatively large sample size (N = 760) of 5-year-old Bulang children, which was about half of the total number 5-year-old Bulang children (estimated to be 2,000) in Yunnan. Therefore a purposeful sampling method used to select the 2 districts where the majority of the Bulang lived is a pragmatic approach for recruitment of participants. Since some children do not attend kindergarten or preschool, it is essential to go to towns and villages to invite children for this survey. The involvement of the local authorities and the support from leaders and elders is crucial for the success of this survey. Multi-stage sampling is generally considered a more accurate cluster sampling for the sample size. This method is also useful when a complete list of all children of the population does not exist or cannot be obtained. In multi-level cluster sampling, representative clusters are chosen and children within the chosen cluster are sampled. This is a cost-effective and efficient method of finding an adequate sample population. However, children selected using the multi-stage cluster sampling method may not be as representative as those selected through random sampling. Another limitation of this study is that it did not assess whether the participating school represented the socioeconomic distribution of the study population. The diverse geographical settlement of Bulang people makes the multi-stage and cluster sampling method an appropriate sampling method. The sampling was performed at 2 stages (district and county) only and further stratification into the kindergarten and preschool was not performed because we needed to invite all kindergartens and preschools to achieve the large sample size.

This study had a large sample size and a high response rate. The support from the local government and the good rapport developed through pre-survey visits to kindergartens were likely two major reasons for the satisfactory outcome. However, the study might not be representative because the sample selection process did not accurately represent the socioeconomic strata in the community. Furthermore, accounts were given by kindergarten children who declined participating in the study. It also did not provide information on village children who did not turn up for the survey.

A simple and easily understood questionnaire was used, and this is one of the ways to guarantee the quality of the study. However, some questions, such as the questions asking about snacking on sweets daily and having bottle feeding before sleep could be too general for inference. This study kept these questions because they had been used in previous studies, and therefore facilitated the comparisons. Nevertheless, more depth is definitely in need if the study aims to elaborate on these factors as possible underlying reasons for the serious caries situation.

A literature review reported that the dentist-to-population ratio in China is around 1:100,000 [5]. The great shortage of dentists in China, especially in rural areas, is likely to be one of the main reasons for the lack of dental treatment. As professional topical fluoride therapy is not available, fluoride toothpastes are therefore the main source of fluoride for these children. However, this survey found that more than half of the Bulang children did not brush their teeth each day. This highlighted the importance of promoting tooth brushing with fluoride-containing toothpaste. Water supply to many villages is inadequate. Children living in villages still lack the clean water that they need to brush their teeth. Integrating oral health promotion into general health care in this population is essential. This study also found that many children had clinical consequence of caries disease. More than one-third of the children had signs of odontogenic infection, which can cause pain and spread infection. Caries can also affect children’s general health, nutrition, growth and quality of life.

The highest caries rate was found in the maxillary incisors of these Bulang children. This observation is consistent with general theories of caries development in children [2]. Our results also showed that the caries rate of posterior teeth was significantly higher in children with caries on their maxillary anterior teeth, and this corroborates with other studies [10,15]. Therefore, children with caries on their maxillary anterior teeth should be taken into consideration upon examination as this can indicate a higher caries rate on the posterior teeth. This study found that children in villages had fewer caries than their counterparts in towns. This finding is contrary to the finding of some surveys conducted in China [21,22]. It is plausible that many Bulang children living in towns had greater access to sweet snacks and hence suffered from a higher caries rate. This study suggested that it is crucial to promote oral health among Bulang children and their parents living in towns. It is also necessary to prevent caries in Bulang children who live in rural, mountainous areas because epidemiological studies show that caries experience of children living in rural areas has soared since the 1980s [21]. This phenomenon of “caries as a disease of civilization” is observed in western countries [23].

The prevalence of caries among the Bulang children is similar to that of the Dai ethnic minority children [15]. This may be because they share living conditions and lifestyles. Their caries experience and prevalence are lower than in they are Cambodia [11], and this could be related to the social, cultural and geographical differences between the two countries. The dentist-to-population ratio is low in China, but higher than it is in Cambodia. Moreover, China has assistant dentists to serve the community, but Cambodia does not [11]. One study has reported that children in remote and mountainous areas of Myanmar had few caries [13]. This may also be related to the socioeconomic and geographical differences between Myanmar and China. In addition, most children in Myanmar do not eat snacks and have a simple, non-cariogenic diet [13].

Recent data indicate that the relationship between sugar consumption and dental caries is not as strong as it was in the pre-fluoride era [24]. Nevertheless, high caries experience was found in Bulang children who consumed sweet snacks each day. The reasons may be the inadequate use of fluoride agents for caries prevention and poor oral hygiene. In contrast to other studies, this study found that the education level of parents was negatively correlated with caries in their children [10,11,13]. This may be because the great majority of Bulang people are poor, and they do not have the chance to learn about oral hygiene. A high caries experience was found among children who visited the dentist in the last year, which was also reported in other studies [10,15]. Because access to dental services is difficult, with the high prevalence of caries, it is plausible that the visit to address the caries was due to associated caries pathology.

## Conclusion

In this survey, the caries prevalence and experience of 5-year-old Bulang children in Yunnan, China was high. Their oral hygiene was poor. Most of the dental caries were left untreated and more than one-third of them had odontogenic infection. The caries experience was associated with snacking habits, dental visit habits and oral hygiene status.

## Competing interests

The authors declare that they have no competing interests.

## Authors’ contributions

SZ performed the survey and ECML, JL and CHC supervised this work. The 4 authors contributed equally to preparation of the manuscript. All authors read and approved the final manuscript.

## Authors’ information

Dr. Shinan Zhang is a PhD student in the Faculty of Dentistry, Prof Edward C.M. Lo. Dr. Juan Liu and C.H. Chu are the supervisors of Dr. Zhang.

## Pre-publication history

The pre-publication history for this paper can be accessed here:

http://www.biomedcentral.com/1472-6831/14/16/prepub

## Supplementary Material

Additional file 1 Questionaire. Click here for file
