# Supplementary material for: Dental caries status of Bulang preschool children in Southwest China
Source: BMC Oral Health. 2014 Mar 4;14:16. doi: 10.1186/1472-6831-14-16 (PMC3946148; doi:10.1186/1472-6831-14-16)
Supplement: Additional file 1 — Questionaire. [file 1472-6831-14-16-S1.docx]

Case No:

**Oral health survey of Bulang preschool children in Yunnan**

1. Child name：________________ Class：________________

2. Contact number：________________

3. Child gender：□_1_ Male　□_2_ Female

4. Where does the child live?

□_1_ Town □_2_ Village

5. Are parents the main care taker of the child?

□_1_ Yes □_2_ No

6. Is your child still sleep with a bottle with milk/sweet drink?

□_1_ Yes □_2_ No

7. Does your child brush his/her teeth daily?

□_1_ Yes □_2_ No

8. Does your child take snacks daily?

□_1_ Yes □_2_ No

9. Did your child visit a dentist in the last 12 months?

□_1_ Yes □_2_ No

10. Father’s education level：

| □_1_ Below secondary | □_2_ Secondary | □_3_ Tertiary or above |
| --- | --- | --- |

11. Mother’s education level：

| \| □_1_ Below secondary \| □_2_ Secondary \| □_3_ Tertiary or above \| \| --- \| --- \| --- \| |  |
| --- | --- | --- | --- | --- |

~ The End ~
